# Diabetes-Related Stigma and Interpersonal Distress Among Adults with Diabetes: A Cross-Sectional Study of Family, Workplace, and Healthcare Settings

**DOI:** 10.3390/healthcare14121705

**Published:** 2026-06-15

**Authors:** Majed M. Aljabri, Bandar S. Alharbi, Endale Alemayehu Ali

**Affiliations:** 1 Community and Psychiatric Mental Health Nursing Department, College of Nursing, King Saud University, Riyadh 12375, Saudi Arabia; banalharbi@ksu.edu.sa; 2 Department of Public Health and Primary Care, KU Leuven, Kapucijnenvoer 33, 3000 Leuven, Belgium

**Keywords:** diabetes stigma, diabetes distress, interpersonal distress, psychosocial factors, Saudi Arabia, chronic disease, social determinants of health

## Abstract

**Background**: Diabetes-related stigma is an underrecognized psychosocial factor that may contribute to emotional burden among individuals with diabetes. In Saudi Arabia, where the prevalence of diabetes is among the highest globally, limited evidence exists on how stigma across different social contexts influences interpersonal diabetes distress. We aimed to assess the association between diabetes-related stigma and interpersonal diabetes distress and to determine whether these associations differed across family, workplace, and healthcare stigma domains among adults with diabetes in Saudi Arabia. **Methods**: This cross-sectional study analyzed survey data collected from 438 patients with diabetes. Diabetes-related stigma was measured using an adapted 12-item diabetes stigma scale covering family, workplace, and healthcare domains, while interpersonal diabetes distress was assessed using the Interpersonal Distress subscale of the Diabetes Distress Scale (DDS). The relationships between stigma and distress were estimated using multiple linear regression analysis adjusted for age, gender, education level, years since diagnosis, and presence of complications. **Results**: Participants reported moderate levels of stigma (mean: 2.50, SD: 1.08) and interpersonal distress (mean: 2.31, SD: 1.23). Higher stigma scores were strongly associated with greater interpersonal distress (β = 0.57, 95% CI: 0.48 to 0.66). Domain-specific analysis showed that workplace (β = 0.26, 95% CI: 0.10 to 0.42) and healthcare stigma (β = 0.23, 95% CI: 0.07 to 0.38) were significantly associated with distress, while family stigma was not. Individuals with diabetes complications had higher distress (β = 0.49, 95% CI: 0.25 to 0.73). No evidence of effect modification by gender or education was observed. Spline models confirmed a positive and strengthening association at higher levels of stigma. **Conclusions**: Diabetes-related stigma is a strong and consistent factor associated with interpersonal diabetes distress in Saudi Arabia, with workplace and healthcare stigma demonstrating the strongest associations. These findings highlight the importance of addressing stigma within both social and healthcare environments and suggest that stigma reduction strategies may help alleviate the psychosocial burden associated with diabetes.

## 1. Introduction

The condition of diabetes mellitus poses a critical problem of public health, with a rapidly increase in both high and middle-income nations [1,2]. In the Middle East as a whole and especially in Saudi Arabia, it can be noted that the prevalence of the disease is relatively very high compared to other parts of the globe, with estimates suggesting that Saudi Arabia ranks second in the region and seventh worldwide [3].

There are serious psychological impacts associated with diabetes besides its metabolic implications. The emergence of diabetes distress (DD) represents an important concept in highlighting the difficulties encountered in managing a chronic illness like diabetes, which include the fear of experiencing complications, the burden of diabetes treatments, and the social pressures related to the illness [4]. Based on global literature, it has been observed that clinically significant DD affects between 20% and above 50% of patients with diabetes [5,6,7]. In Saudi Arabia, the results obtained from the studies conducted show comparable levels of DD among patients [8]. Higher levels of DD are associated with poor glycemic control, low adherence to diabetes medications, and complications related to diabetes [9,10].

Several studies assessed the impact of stigma as an important yet often ignored factor influencing outcomes for people living with diabetes. Stigma related to diabetes includes negative stereotyping, discrimination, and social isolation towards people diagnosed with diabetes [11]. Such attitudes may be fueled by misunderstandings regarding the origins of diabetes, primarily the notion that diabetes results from individual decisions alone. People living with diabetes will experience judgmental attitudes from their family members, employers, and even physicians, leading to poor self-management and mental distress [12].

The relationship between stigma and psychological outcomes can be understood through contemporary stigma frameworks, which conceptualize stigma as a multidimensional social process involving enacted stigma, anticipated stigma, and internalized stigma [13,14]. These mechanisms may adversely affect emotional well-being by reducing self-esteem, increasing social isolation, disrupting supportive relationships, and limiting engagement with healthcare services. Individuals who perceive stigma may anticipate negative judgments from others, leading to concealment of their condition and withdrawal from social interactions. Despite increasing recognition of these pathways, limited evidence exists regarding how stigma experienced in different social contexts influences interpersonal diabetes distress, particularly in Middle Eastern populations.

Stigma becomes more relevant in Saudi Arabia where there are strong social and cultural norms concerning individual autonomy, family responsibilities, and social reputation. The pressure of meeting cultural standards with regard to health and chronic diseases might lead to hiding one’s condition and internalizing stigma. A study in Saudi Arabia showed that there was a moderate level of stigma among people with diabetes in terms of blaming and making judgments about people living with diabetes [15]. Also, stigma negatively affects self-management and emotion regulation.

Though the problems are well recognized, there still appears to be limited empirical research into the issue in Saudi Arabia, with the existing literature focusing mostly on attitudes towards diabetes generally or on self-stigma in people with diabetes, often using one combined scale [11]. Relatively few studies have considered the ways in which stigma functions in different social environments, including family, work place, and healthcare environments. The lack of such studies is particularly crucial as the phenomenon of stigma is not a single one and could vary depending on the specific context in which social interaction takes place. On the other hand, although diabetes distress has been studied in Saudi Arabia, it is relatively rarely measured in the course of medical practice in the country and its predictors require further study [16]. Clinical factors associated with diabetes such as glycemic control, complications, and diabetes management have received much more attention in previous studies compared to non-clinical and social aspects, especially interpersonal aspects, which is relevant to the collectivist culture of Saudi Arabia.

The interrelationship between stigma and distress in people with diabetes still lacks empirical exploration. Recent studies suggest that stigma might act as a psychosocial stressor, increasing emotional burden and interfering with coping mechanisms. However, most previous investigations focused on assessing stigma and distress individually, without considering these variables together in an integrated manner. There are limited studies that estimate the contribution of stigma to interpersonal distress in relation to other socio-demographic and clinical variables [17].

The aim of this study is to quantify the magnitude of the association between diabetes-related stigma and interpersonal diabetes distress in people suffering from diabetes in Saudi Arabia. In particular, our study focuses on measuring both general stigma and specific stigmas related to the family setting, workplace environment, and healthcare settings, after controlling for various sociodemographic and clinical characteristics.

## 2. Methods

### 2.1. Study Design and Data Source

This cross-sectional study was conducted in Saudi Arabia using a structured questionnaire administered among adults with diabetes attending four primary healthcare centers located in different regions of Riyadh. Data collection took place between 7 and 15 April 2026. Eligible participants were adults with a physician-confirmed diagnosis of diabetes who were able to understand the study information and complete the questionnaire independently. Recruitment was conducted using a convenience sampling approach. Individuals attending the participating healthcare centers were invited to participate through study information sheets containing a QR code that linked directly to the online questionnaire. The QR code was also displayed on notice boards within the healthcare facilities to maximize visibility and participation.

The survey was administered using Google Forms and included questions on socio-demographic characteristics, clinical history, diabetes-related stigma, and diabetes distress. Participation was voluntary, and electronic informed consent was obtained before survey initiation. A total of 489 participants completed the survey. For the present analysis, respondents with incomplete information on the main exposure, outcome, or covariate variables were excluded, resulting in a final analytic sample of 438 (90%) participants. Observations with missing responses were removed before data processing and analysis.

### 2.2. Measures

#### 2.2.1. Diabetes-Related Stigma

Diabetes-related stigma was assessed using a 12-item scale adapted from previously validated stigma instruments [18,19]. In this study, the scale captured perceived stigma across three domains including family, workplace, and healthcare domains.

The family domain included items assessing perceived anger, blame, reduced respect, and attribution of responsibility for the condition. The workplace domain captured perceptions of discrimination, limited promotion opportunities, assignment of less important tasks, and perceptions of reduced competence. The healthcare domain assessed experiences related to provider frustration, blame, perceived lower quality of care, and perceptions of being viewed as noncompliant.

Each item was measured on a 7-point Likert scale ranging from “very unlikely” (1) to “very likely” (7), reflecting the perceived likelihood of experiencing stigmatizing attitudes or behaviors. Domain-specific scores were calculated as the mean of the four items within each domain, resulting in family stigma, workplace stigma, and healthcare stigma scores. A total stigma score was calculated as the mean of all 12 items, with higher scores indicating greater perceived stigma.

The internal consistency of the instrument was evaluated using Cronbach’s alpha. The overall stigma scale demonstrated excellent reliability (α = 0.94).

#### 2.2.2. Interpersonal Diabetes Distress

Interpersonal diabetes distress was assessed using the interpersonal distress items of the Saudi Arabian Diabetes Distress Scale (SADDS-17), the Arabic version of the Diabetes Distress Scale (DDS-17) [20] originally developed by Polonsky et al. [21,22]. The DDS-17 is a widely used instrument that measures diabetes-related emotional distress across four domains: emotional burden, physician-related distress, regimen-related distress, and interpersonal distress. The Arabic version (SADDS-17) was translated using a forward-backward translation procedure and psychometrically validated among Saudi adults with type 2 diabetes by Batais et al. [20]. The validation study demonstrated good internal consistency (Cronbach’s α = 0.848), satisfactory test–retest reliability (0.78), and confirmation of the original four-factor structure [22,23].

Each item was rated on a 6-point scale ranging from “not a problem” (1) to “a very serious problem” (6). The interpersonal distress score was computed as the mean of the three items, with higher values indicating greater distress.

The subscale demonstrated good internal consistency (α = 0.88).

#### 2.2.3. Covariates

We adjusted for several socio-demographic variables, including age (categorized as 18–24, 25–34, 35–44, 45–54, 55–64, and ≥65 years), sex (female, male), education level (less than high school, high school, diploma, bachelor’s degree, and graduate), employment status (employed, unemployed, retired, student, and other), and marital status (single, married, divorced, and widowed). Clinical variables included years since diabetes diagnosis (<1 year, 1–5 years, 6–10 years, and >10 years) and the presence of diabetes-related complications (no, yes).

### 2.3. Statistical Analysis

Continuous variables were summarized using means and standard deviations, as well as medians and interquartile ranges, to account for potential skewness in the data. The primary analysis examined the association between diabetes stigma and interpersonal diabetes distress using multivariable linear regression. The outcome variable was the interpersonal distress score, and the main exposure was the total stigma score. Model covariates were selected a priori based on their potential relevance as confounding factors according to previous literature and clinical considerations. The fully adjusted models included age group, sex, education level, years since diabetes diagnosis, and diabetes-related complications. In this study, the term “adjusted” refers to regression estimates obtained after accounting for the effects of these covariates.

Regression coefficients (β) with 95% confidence intervals were reported. Model assumptions, including normality, linearity and homoscedasticity, were assessed through diagnostic plots. Multicollinearity was assessed using generalized variance inflation factors (GVIFs) to account for categorical covariates with multiple degrees of freedom. Adjusted GVIF values (GVIF^(1/(2 × Df))) were examined.

To identify which sources of stigma were most strongly associated with interpersonal diabetes distress, a second multivariable model replaced the total stigma score with the three domain-specific stigma scores (family, workplace, and healthcare stigma). This allowed for the estimation of the independent contribution of each domain while controlling for the same covariates.

We also examined the potential effect modification by sex and education by including interaction terms between stigma and these variables in separate models. Interaction effects were interpreted based on the magnitude and confidence intervals of the interaction coefficients.

To assess potential nonlinear associations between diabetes-related stigma and interpersonal diabetes distress, we fitted regression models incorporating natural cubic splines for the total stigma score. The spline term was specified using three degrees of freedom, allowing for flexible modeling of departures from linearity while avoiding overfitting. Model estimates were adjusted for age group and sex. Results were used as a sensitivity analysis to confirm the robustness of the main findings.

We performed the analysis using R version 4.2.1 [23].

## 3. Results

Diabetes stigma values ranged from moderately low to moderately high in this study population. The overall mean score for stigma was 2.50 (SD 1.08), with a median of 2.58, reflecting a reasonably symmetrical distribution pattern (Table 1). For the individual sub-scales, workplace-related stigma had slightly higher values (mean 2.53), while healthcare-related stigma had slightly lower values (mean 2.47). Interpersonal diabetes distress was also observed at a moderate level, with a mean score of 2.31 (SD 1.23) and a median of 2.00.

The diabetes stigma scale demonstrated excellent internal consistency (Cronbach’s α = 0.94), indicating high reliability across items (Table 2). The interpersonal distress subscale also demonstrated strong reliability (α = 0.88). Item–total correlations were consistently high across all items (r = 0.71–0.83), indicating strong internal coherence within both scales (Supplementary Table S1). Stigma items showed good consistency, while the interpersonal distress items demonstrated slightly higher correlations (r = 0.80–0.83). Item means were comparable across domains, with no indication of poorly performing items.

**Figure 1:**
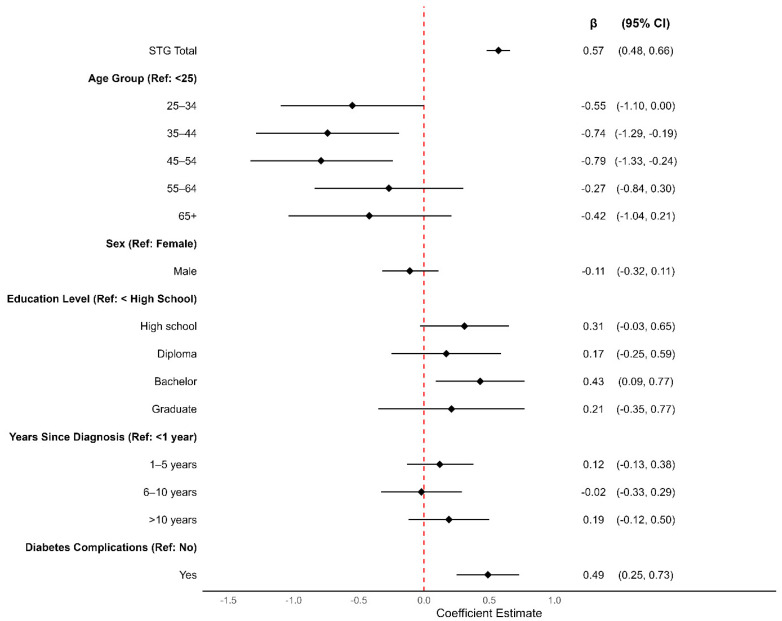
Factors associated with interpersonal diabetes distress in the multivariable linear regression model. Points represent adjusted regression coefficients (β), and horizontal lines represent 95% confidence intervals. The vertical dashed line indicates the null value (β = 0). Statistical significance can be inferred when the 95% confidence interval does not include zero.

We observed a strong and consistent association between diabetes-related stigma and interpersonal distress (Figure 1). A one-unit increase in stigma score was associated with a 0.57 increase in interpersonal distress (β = 0.57, 95% CI: 0.48 to 0.66). There was an inverse association between age group and interpersonal distress. Compared with participants aged 18–24, those aged 25–34 had lower distress (β = −0.55, *p* = 0.048). The effect was stronger in ages 35–44 (β = −0.74, *p* = 0.009) and 45–54 (β = −0.79, *p* = 0.005). No significant association was observed for ages 55–64 or ≥65. No significant association was found for sex. Males showed slightly lower scores than females, but the effect was small and not significant (β = −0.11, *p* = 0.335). There was mixed pattern for education. Compared with participants in the lowest education category, those with a bachelor’s degree reported higher levels of interpersonal distress (β = 0.43, 95% CI: 0.09 to 0.77), whereas no significant associations were observed for the remaining education categories. Years since diabetes diagnosis was not significantly associated with interpersonal distress. In contrast, diabetes-related complications were associated with higher interpersonal distress. Participants reporting complications had distress scores that were 0.49 units higher than those without complications (β = 0.49, 95% CI: 0.25 to 0.73).

**Figure 2:**
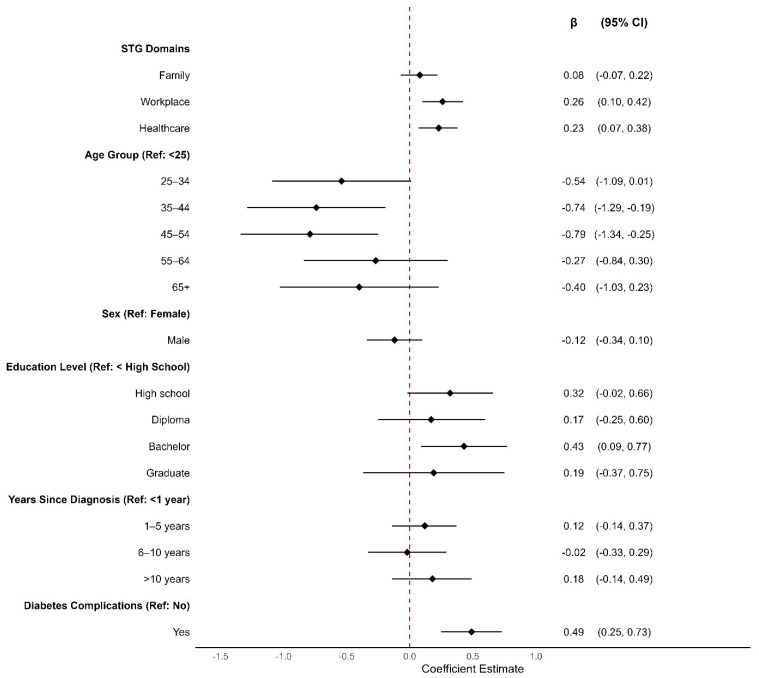
Associations between diabetes stigma domains and interpersonal diabetes distress from the multivariable linear regression model. Points represent adjusted regression coefficients (β), and horizontal lines represent 95% confidence intervals. The vertical dashed line indicates the null value (β = 0). Positive coefficients indicate higher interpersonal diabetes distress relative to the reference group or per unit increase in the corresponding stigma domain score, whereas negative coefficients indicate lower distress. Statistical significance can be inferred when the 95% confidence interval does not include zero.

The association between stigma domains and interpersonal diabetes are presented in Figure 2. Our findings showed that workplace and healthcare sigma showed significant associations with higher interpersonal diabetes distress. A one-unit increase in workplace stigma was associated with a 0.26 increase in distress (β = 0.26, 95% CI: 0.10 to 0.42). Healthcare stigma showed a similar effect (β = 0.23, 95% CI: 0.07 to 0.38). In contrast, family stigma was not associated with interpersonal distress (β = 0.08, *p* = 0.318).

Estimates for sociodemographic and clinical covariates were consistent with the main model, indicating that the observed associations are robust to different parameterizations of stigma. Our finding highlighted that stigma experienced in workplace and healthcare settings, rather than within the family, drives interpersonal diabetes distress.

We did not observe effect modification by sex and education level (Supplementary Table S2). The association between stigma and interpersonal distress remained positive and consistent across groups. Interaction terms for sex and education were small and not statistically significant, indicating no meaningful differences in the stigma–distress relationship based on sex or educational level.

The spline model shows a strong positive association between diabetes stigma and interpersonal distress across the entire range of stigma (Supplementary Table S3). We found statistically significant association for all spline terms (*p* < 0.001), indicating that the relationship is not purely linear. The increasing magnitude of the spline coefficients suggests that distress rises more steeply at higher levels of stigma. This supports a nonlinear pattern, where the impact of stigma becomes stronger as stigma increases. Despite this nonlinearity, the direction of the association remains consistent. There is no evidence of a threshold or reversal. There was consistent finding for age. Individuals aged 25–54 reported significantly lower distress compared to the youngest group. No clear differences were observed for older age groups.

We performed a series of model diagnosis check. Multicollinearity was assessed using Generalized Variance Inflation Factors and found that there is no problem of multicollinearity (Supplementary Table S4). Residuals vs fitted values show a random scatter around zero with no clear pattern (Supplementary Figure S1, top left panel). This suggests that the linearity assumption is reasonable and there is no strong model misspecification. The Q–Q plot shows slight deviations at the tails, which is expected with real data (top right panel). Given the sample size of 438, these deviations are not concerning. The scale–location plot indicates relatively constant spread of residuals across fitted values (bottom left panel). There is no clear funnel shape, suggesting no major heteroscedasticity.

The leverage plot does not show influential observations with both high leverage and large residuals (bottom right panel).

## 4. Discussion

### 4.1. Main Findings

This study provides empirical evidence on the association between diabetes-related stigma and interpersonal diabetes distress. Higher levels of perceived stigma were consistently associated with greater interpersonal diabetes distress, even after adjustment for socio-demographic and clinical characteristics. Importantly, these associations differed across stigma domains; stigma experienced in workplace and healthcare settings showed significant association, whereas family-related stigma did not retain significance. The association between stigma and distress was robust across subgroups, with no evidence of effect modification by gender or education. In the nonlinear model, we showed that the impact of stigma may intensify at higher levels, indicating a dose–response pattern rather than a simple linear association.

### 4.2. Comparison to Previous Studies

The findings of this study align with several studies on quantifying stigma as a critical psychosocial determinant of diabetes outcomes. Jane Speight et al. has shown that diabetes stigma is linked to emotional distress, reduced self-management, and poorer quality of life [24]. A study in Australia also found a significant association between stigma and diabetes distress and confidence in a diabetes self-care scale [25]. A meta-analysis of 19 studies with 12,777 participants clearly showed that a strong association between stigma and psychological distress among people with diabetes [26].

The role of diabetes stigma in creating interpersonal diabetes distress involves a series of interrelated psychosocial processes. The current study suggests that stigma has an enduring association with psychological distress experienced by people with diabetes, including both direct relationships and those mediated by other factors [26]. Internalized stigma is a central pathway in which the individual feels embarrassed and ashamed to the extent that their self-esteem is lowered, and psychological burdens are increased; this is correlated with worse psychological consequences and quality of life [27]. Stigma reduces self-efficacy and perceived control, both of which are important elements for diabetes self-management, and causes interpersonal diabetes distress [28]. At the same time, stigma interferes with social support systems by either creating conflicts in interpersonal relationships or encouraging the individual to hide their diagnosis, causing loneliness and lack of emotional support. Perceived stigma may become internalized, resulting in feelings of shame, self-blame, and reduced self-worth. Stigma may also be anticipated, leading individuals to conceal their condition, avoid social interactions, and reduce help-seeking behaviors. Furthermore, stigmatizing experiences can disrupt social support networks and contribute to chronic stress, ultimately increasing emotional burden and interpersonal difficulties.

In this study we also assessed the specific aspects of stigma related to diabetes, instead of considering stigma as one-dimensional, as was typically done in past studies [26]. Our findings showed that stigma in the workplace setting and stigma in the healthcare setting were significantly associated with interpersonal distress, while stigma in relation to family members did not appear to be significant. This finding is consistent with emerging evidence that stigma in institutional and formal settings tends to have a stronger impact on psychological outcomes than stigma within close social networks [18,29]. In workplace contexts, individuals with chronic conditions often face subtle discrimination, reduced career opportunities, and concerns about being perceived as less competent, all of which can contribute to stress and interpersonal strain [30,31]. Similarly, stigma in healthcare settings, particularly perceived blame or lack of empathy from providers, has been shown to increase diabetes distress and reduce patient engagement with care [26]. In contrast, the lack of significant association for family-related stigma may reflect the buffering role of family support, especially in collectivist settings such as Saudi Arabia, where family networks often provide emotional and practical support that mitigates the negative effects of stigma. The nonlinear analysis provides further insight into the association between stigma and distress. The increase in size of the spline coefficients shows that as the amount of stigma increases, so does the intensity of the relationship between stigma and distress. It implies a kind of cumulative effect that means that greater exposure to stigma leads to exponentially higher levels of distress.

The association between diabetes complications and higher interpersonal distress is consistent with existing literature [26,32]. Individuals with complications may experience greater physical limitations, increased treatment burden, and heightened awareness of disease severity, all of which can contribute to distress. However, the persistence of the stigma effect after adjusting for complications indicates that stigma operates independently of clinical severity. This reinforces the need to address psychosocial factors alongside biomedical management.

While our study focused on stigma and interpersonal distress, it is also important to that many individuals with diabetes demonstrate substantial psychological resilience despite the challenges associated with disease management. Resilience refers to the ability to adapt positively to adversity and maintain psychological well-being in the face of chronic health conditions [33]. Evidence from Middle Eastern and Arab populations suggests that resilience, self-efficacy, adaptive coping strategies, and strong family support can protect against diabetes-related distress and improve quality of life [34]. Individuals with higher resilience levels tend to report better emotional adjustment, greater treatment adherence, and lower levels of diabetes-related burden. These protective factors may partially buffer the negative effects of stigma and help explain why not all individuals exposed to stigmatizing experiences develop significant psychological distress.

This study addresses several important gaps in the literature. First, it provides evidence from Saudi Arabia, a region with a high diabetes prevalence but limited research on stigma and psychosocial outcomes. Second, it moves beyond simple descriptive analyses by applying multivariable, domain-specific, and nonlinear models. Third, it focuses on interpersonal distress, a dimension that is highly relevant but underexplored. Together, these contributions enhance our understanding of the mechanisms linking social experiences to emotional outcomes in diabetes.

The findings have important implications for practice and policy. Interventions aimed at reducing diabetes distress should incorporate strategies to address stigma at multiple levels. In the workplace, this may include awareness campaigns, anti-discrimination policies, and supportive work environments. In healthcare settings, training programs for providers can promote empathetic communication and reduce stigmatizing attitudes. Public health initiatives can also play a role in challenging misconceptions about diabetes and reducing blame-oriented narratives.

### 4.3. Strengths and Limitations

This study has several potential strengths. A major strength lies in the multidimensional assessment of diabetes-related stigma. By disaggregating stigma into family, workplace, and healthcare domains, the analysis moves beyond the conventional approach of treating stigma as a single construct. This allowed for the identification of context-specific effects and revealed that stigma is not uniformly experienced or impactful. The study also focuses specifically on interpersonal diabetes distress, a dimension that captures relational difficulties and social functioning. The use of multivariable models with adjustment for important socio-demographic and clinical factors reduces confounding and supports the robustness of the observed associations. Additional analyses, including domain-specific models, interaction terms, and nonlinear modeling, provide a comprehensive assessment of the relationship and allow for the evaluation of heterogeneity and dose–response patterns. The study also provides context-specific evidence from Saudi Arabia, a setting with a high burden of diabetes but limited research on stigma and psychosocial outcomes.

However, several limitations should be considered when interpreting the findings. The use of cross-sectional design limits the ability to analyze causal effects and trends over time. The study employed a convenience sampling strategy using a voluntary QR-code-based survey, which may have introduced self-selection bias. Individuals who chose to participate may differ systematically from those who did not participate, particularly with respect to health awareness, digital literacy, or psychosocial experiences. Consequently, the findings may not be fully representative of all adults living with diabetes in Saudi Arabia, and caution is warranted when generalizing the results to other populations. The diabetes stigma instrument was adapted from existing stigma measures and has not undergone formal validation in an independent Saudi population. While the instrument demonstrated excellent internal consistency in the present study, further work is needed to evaluate additional psychometric properties, including construct validity, criterion validity, and factor structure. Although the instruments demonstrated strong reliability, cultural adaptation and validation remain important considerations, particularly when applying scales across different contexts. Although the analyses adjusted for several important socio-demographic and clinical characteristics, the possibility of residual confounding cannot be excluded. Several psychosocial factors known to be associated with both stigma and diabetes distress, including depression, anxiety, social support, self-efficacy, and socioeconomic status, were not assessed in the present study. Consequently, part of the observed association may reflect the influence of these unmeasured factors. Finally, the interpretation of nonlinear effects, while informative, remains exploratory. Although the findings suggest a strengthening association at higher levels of stigma, further study using longitudinal and experimental designs is needed to confirm these patterns and understand underlying mechanisms.

## 5. Conclusions

Our findings showed that diabetes-related stigma is a significant factor associated with interpersonal diabetes distress. The impact varies by context, with workplace and healthcare stigma showing the most pronounced association, while family-related stigma appears less influential. The association persists across demographic and clinical groups and strengthens at higher levels of stigma. These findings highlight stigma as a key psychosocial determinant that should be addressed in both clinical practice and public health strategies to reduce the emotional burden of diabetes.

## Figures and Tables

**Table 1:** Descriptive statistics for diabetes stigma and interpersonal diabetes distress scores among study participants, presented as mean (standard deviation) and median (interquartile range).

Variable	Mean (SD)	Median (IQR)
**Diabetes Stigma (STG)**		
Total Stigma Score	2.50 (1.08)	2.58 (1.42)
Family Domain	2.50 (1.17)	2.50 (1.50)
Workplace Domain	2.53 (1.18)	2.50 (1.50)
Healthcare Domain	2.47 (1.13)	2.50 (1.50)
**Diabetes Distress (DDS)**		
Interpersonal Diabetes Distress	2.31 (1.23)	2.00 (2.00)

**Table 2:** Internal consistency of stigma and interpersonal distress scales.

Scale	Items (n)	Cronbach’s α (95% CI)	Mean (SD)	Average Inter-Item Correlation
Diabetes stigma (STG)	12	0.94 (0.93, 0.95)	2.50 (1.10)	0.57
Interpersonal diabetes distress (DDS)	3	0.88 (0.85, 0.89)	2.30 (1.20)	0.70

## Data Availability

The datasets used in this study are available from the first author on reasonable request.

## Supplementary Materials

The following supporting information can be downloaded at: https://www.mdpi.com/article/10.3390/healthcare14121705/s1, Figure S1: Regression diagnostic plots for the main multivariable model; Table S1: Item-level statistics for the diabetes stigma scale and interpersonal diabetes distress subscale; Table S2: Interaction effects between diabetes stigma and gender and education on interpersonal diabetes distress; Table S3: Nonlinear association between diabetes stigma and interpersonal diabetes distress using restricted cubic spline regression, adjusted for age and gender; Table S4: Generalized Variance Inflation Factors (GVIF) for Model Covariates.
