# Supplementary material for: Diabetes-Related Stigma and Interpersonal Distress Among Adults with Diabetes: A Cross-Sectional Study of Family, Workplace, and Healthcare Settings
Source: Healthcare (Basel). 2026 Jun 15;14(12):1705. doi: 10.3390/healthcare14121705 (PMC13300341; doi:10.3390/healthcare14121705)
Supplement: Supplementary file 1 [file healthcare-14-01705-s001.zip › healthcare-4333413-Supplementary.pdf]

Supplementary Material

Diabetes-Related Stigma and Interpersonal Distress Among Adults with Diabetes: A Cross-Sectional Study of Family, Workplace, and Healthcare Settings

Majed M. Aljabri[1*], Bandar S. Alharbi[1], Endale Alemayehu Ali[2]*

[1]Community and Psychiatric Mental Health Nursing Department, College of Nursing, King Saud University, Riyadh 12375, Saudi Arabia

[2]Department of Public Health and Primary Care, KU Leuven, Kapucijnenvoer 33, 3000 Leuven, Belgium

[*] Shared corresponding author (Email: amajad@ksu.edu.sa; endalestat@gmail.com)

Table S1. Item-level statistics for the diabetes stigma scale and interpersonal diabetes distress subscale.

**Item**	**Item-total correlation (r)**	**Mean**	**SD**
**Diabetes stigma (STG)**			
Family anger	0.74	2.3	1.4
Family blame	0.74	2.7	1.5
Blamed for condition	0.71	2.7	1.5
Less respect	0.78	2.3	1.4
No promotion	0.79	2.4	1.4
Workplace discrimination	0.77	2.5	1.4
Less important tasks	0.78	2.6	1.4
Work incompetence	0.79	2.6	1.4
Provider frustration	0.77	2.4	1.3
Lower quality care	0.77	2.3	1.3
Provider blame	0.74	2.6	1.4
Seen as noncompliant	0.74	2.7	1.4
**Interpersonal diabetes distress (DDS)**			

Lack of family support	0.80	2.2	1.4
People do not understand	0.81	2.4	1.4
Diabetes affects relationships	0.83	2.3	1.4

Table S2. Interaction effects between diabetes stigma and gender and education on interpersonal diabetes distress.

**Variable**	**Gender interaction β (95% CI)**	**Education interaction β (95% CI)**
Intercept	1.32 (0.57, 2.07)	1.57 (0.66, 2.49)
Stigma (total)	0.56 (0.40, 0.72)	0.47 (0.22, 0.71)
Male (vs Female)	-0.23 (-0.78, 0.31)	-0.13 (-0.35, 0.09)
Age (ref: 18–24)		
25–34	-0.56 (-1.12, 0.00)	-0.55 (-1.11, 0.01)
35–44	-0.75 (-1.31, -0.19)	-0.73 (-1.29, -0.17)
45–54	-0.74 (-1.29, -0.20)	-0.73 (-1.28, -0.19)
55–64	-0.21 (-0.78, 0.35)	-0.19 (-0.76, 0.37)
≥65	-0.31 (-0.92, 0.30)	-0.31 (-0.92, 0.29)
Education (ref: Primary or less)		
High school	0.19 (-0.15, 0.53)	-0.10 (-0.95, 0.75)
Diploma	0.10 (-0.33, 0.53)	0.14 (-0.92, 1.20)
Bachelor	0.33 (-0.01, 0.67)	-0.21 (-1.04, 0.62)
Graduate	0.22 (-0.36, 0.79)	-0.55 (-1.97, 0.87)
Interaction terms		
Stigma × Male	0.04 (-0.15, 0.24)	—
Stigma × High school	—	0.11 (-0.19, 0.40)
Stigma × Diploma	—	-0.02 (-0.39, 0.34)
Stigma × Bachelor	—	0.21 (-0.08, 0.50)
Stigma × Graduate	—	0.30 (-0.22, 0.82)

Table S3. Nonlinear association between diabetes stigma and interpersonal diabetes distress using restricted cubic spline regression, adjusted for age and gender.

**Predictor**	**β (95% CI)**	**SE**	**p-value**
(Intercept)	1.98 (1.40, 2.57)	0.30	< 0.001
STG Total (Spline)			
Term 1	1.41 (0.88, 1.93)	0.27	< 0.001
Term 2	3.12 (2.39, 3.85)	0.37	< 0.001
Term 3	2.70 (1.77, 3.63)	0.47	< 0.001
Age Group (Ref: <25)			
25–34	-0.57 (-1.12, -0.01)	0.28	0.047
35–44	-0.76 (-1.31, -0.20)	0.28	0.007
45–54	-0.78 (-1.32, -0.24)	0.28	0.005
55–64	-0.28 (-0.84, 0.28)	0.28	0.325
65+	-0.44 (-1.02, 0.15)	0.30	0.147
Gender			
Male (Ref: Female)	-0.08 (-0.29, 0.14)	0.11	0.481

Table S4: Generalized Variance Inflation Factors (GVIF) for Model Covariates

**Predictor**	**GVIF**	**Df**	**$GVIF^{1/(2\times Df)}$**
STG Total	1.06	1	1.03
Age	1.80	5	1.06
Gender	1.11	1	1.06
Education Level	1.48	4	1.05
Years Since Diagnosis	1.67	3	1.09
Diabetes Complications	1.10	1	1.05

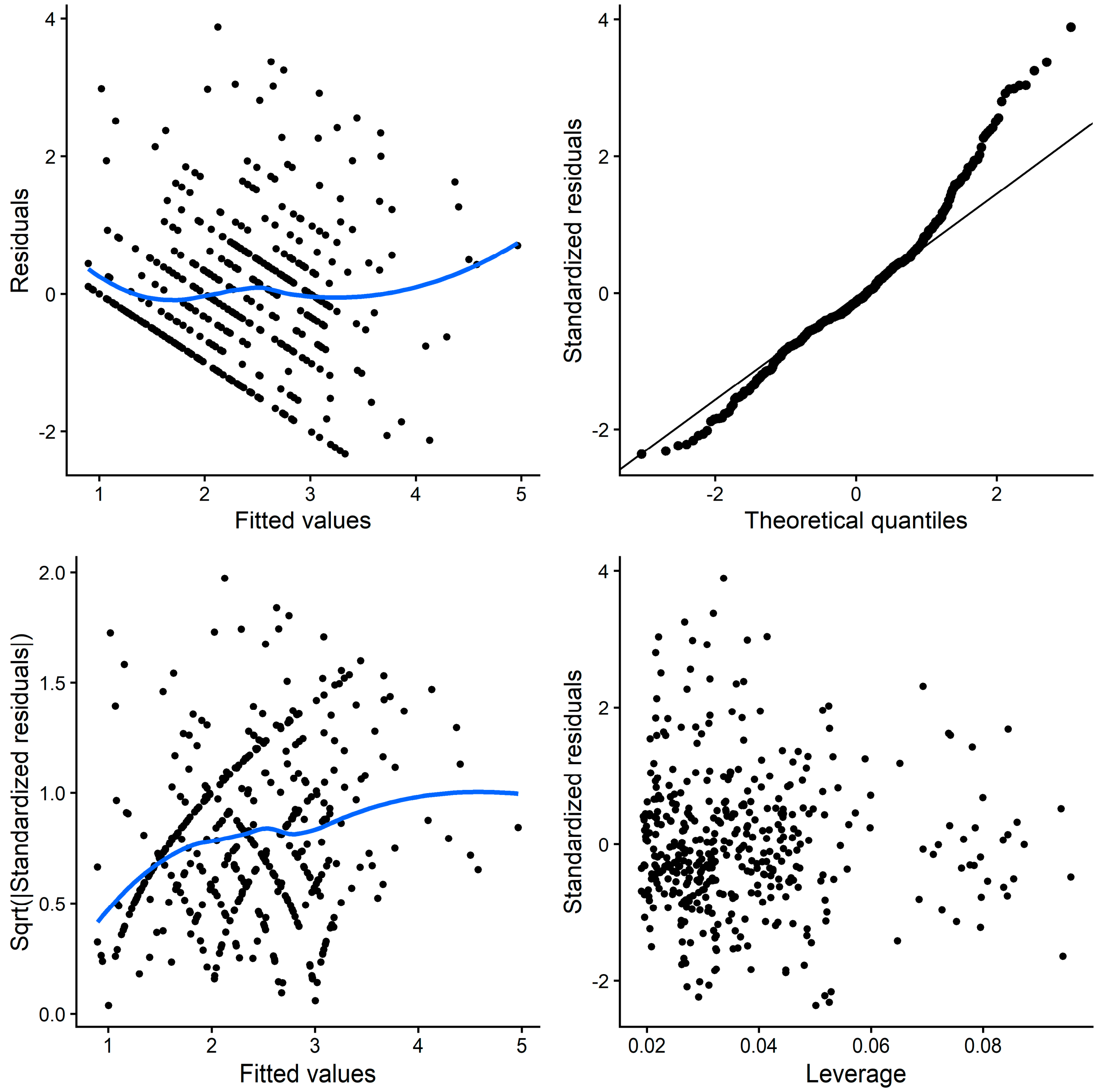

Figure S1: Regression diagnostic plots for the main multivariable model
